# Cognitive reserve can impact trajectories in ageing: a longitudinal study

**DOI:** 10.1007/s40520-025-03000-z

**Published:** 2025-03-17

**Authors:** Sonia Montemurro, Raffaella Ida Rumiati, Veronica Pucci, Massimo Nucci, Sara Mondini

**Affiliations:** 1 Department of Philosophy, Sociology, Education and Applied Psychology (FISPPA), University of Padua, Padua, Italy; 2 Human Inspired Technology Research Centre - HIT, University of Padua, Padua, Italy; 3 Neuroscience, SISSA, Trieste, Italy; 4 Department of General Psychology, University of Padua, Padua, Italy; 5 IRCCS San Camillo Hospital, Venice, Italy

**Keywords:** Clinical trajectories, Ageing, Cognitive reserve, Neurodegeneration

## Abstract

Trajectories of decline from healthy aging to dementia are not linear; they may range from a more attenuated to a more accelerated cognitive worsening. This longitudinal study investigates the impact of Cognitive Reserve (CR) on task performance in a cohort of 117 individuals (mean age 74.89; SD = 7.43) who experienced difficulties in everyday life and referred to a neuropsychology unit for cognitive assessment. Only participants assessed three times were included in the study. CR was estimated for each participant based on educational level and occupational complexity. Results revealed a general decline in global cognitive performance over time, in particular from the second to the final assessment (mean years = 2.95 ± 2.11). The two groups (high CR vs. low CR) did not differ for sex or age across assessments. Individuals with high CR not only exhibited greater cognitive efficiency at baseline—consistent with Stern’s (2009) construct—but also maintained a more stable cognitive state over time compared to those with low CR. Post-hoc analyses confirmed that while the two groups did not differ in task performance between the first and the second assessment, they significantly differed from the second to the third. Individuals with high CR (even those who were diagnosed with a major neurocognitive disorder) preserved their cognitive profile whereas the profile of those with low CR declined significantly. This study demonstrates that cognitive trajectories are shaped by CR and that, despite the plausible progression of brain deterioration, individuals with high CR may not experience an accelerated cognitive deterioration. Instead, they may exhibit a more gradual decline and sustain cognitive functioning for a longer period despite age-related changes.

**Supplementary Information:**

The online version contains supplementary material available at 10.1007/s40520-025-03000-z.

## Introduction

Maintaining cognitive health and a high quality of life in the elderly is vital for both socio-economic and individual reasons. The concept of cognitive and brain resilience [1], which is argued to attenuate the physiological age-related decline, is useful when trying to understand the complexity of age-related changes and the factors contributing to successful ageing. While many behaviours such as smoking, alcohol consumption, unhealthy eating and low physical exercise are known as risk factors for cognitive decline, the key elements of resilience, such as education and type of occupation, are those that more directly build Cognitive Reserve (CR). Educational attainment and working activities (on a continuum of complexity degrees) requires cognitive resources and at the same time it enhances them [see 2] thus preparing a foundation of knowledge and skills.

Based on the construct of CR, individuals vary in their ability to cope with ageing or brain-related diseases [2] to the extent that, in some clinical conditions, such knowledge and skills may become protective in later life and also in the later stages of their disease [3, 4]. Nowadays this is well recognized [5] and it suggests that the wider the range of cognitively stimulating activities someone engages in, the greater their reserve in later years. According to the dominant perspective in the framework of CR, individuals with high CR would experience possible brain deterioration later in life than individuals with low CR. The former may compensate through a set of alternative strategies, which would be parallelled by the recruitment of alternative networks or structures in the brain [6, 7].

Since the earliest formulation of Stern’s CR theory, it has been assumed that, at least from the onset of dementia, high CR individuals would face a more rapid decline over time than those with low CR [2]. More specifically, individuals with high CR would cope with age-related pathology for longer and, albeit maintaining cognitive efficiency over time, their accumulated pathology would unavoidably manifest itself at some point as accelerated deterioration. Such acceleration has been hypothesized to occur either before or after the diagnosis of dementia [6, 7] suggesting that, generally, an increasingly steep and non-gradual decline should be expected in people with high CR, but not in people with low CR [2, 8]. This pattern was repeatedly proposed later in further studies [9] in which a model was developed indicating that patients with Alzheimer’s Disease (AD) with higher CR have more rapid rates of decline. The results of a further and more recent study [10] have shown that, among Aβ-positive individuals, greater CR can be associated with blended clinical progression in the pre-dementia stages of AD, but with a steeper cognitive decline after the onset of AD. Trajectories of changes associated with CR have also been investigated in a study assessing non-pathological older adults. The authors addressed the role of education in predicting cognitive functioning, analyzing over a period of 8 years the data of 5642 older adults aged over 60 and found that education did not have the effect of slowing down the decline.

However, the results from further studies [11] have been inconsistent with the above findings and Stern’s theory [2], showing that AD patients with higher CR can experience a significantly attenuated decline of cognitive functioning, especially with executive functions. In this same context, another study [12] suggested that higher CR may lead to maintaining cognitive efficiency over time in ageing individuals with brain atrophy. In their study, Bettcher and colleagues [12] operationalized CR as “residual cognitive abilities not accounted for by demographic and brain variables”. However, life-experience proxies that are known to influence CR, such as occupational activities throughout the lifespan [5, 13], were not considered in their study. To date, CR has mainly been used to explain the complex relationship between the brain and overt cognitive state [5], but how the different levels of CR can influence ageing trajectories over time has yet to be clarified. Thus, our aim was to provide more evidence of the effect of CR (i.e., including both education and occupational attainment) in test performance over time by analyzing a dataset of consecutive older adults who were repeatedly assessed for neuropsychological functioning.

## Methods

From a cohort of 4638 individuals, 3055 (1997 females) with age-related cognitive complaints or overt decline were included in the study, while individuals with severe psychiatric comorbidities or other critical conditions (e.g., recent major vascular events; Parkinson’s disease; Multiple sclerosis) were excluded. The medical and neurological history of each participant was collected, and information from Computerized Tomography or Magnetic Resonance Imaging was taken into account for the diagnosis. All information was collected by consulting medical records or interviews. The final cohort consisted of individuals with different levels of age-related cognitive decline, enrolled between 2002 and 2016 at a Hospital (Neuropsychological Service) in the North of Italy. All participants who had undergone three assessments were considered in the present analysis.

During the neuropsychological evaluation, all participants were assessed on a one-hour-long battery of tests that included the MMSE (Mini-Mental State Examination) [14] a cognitive screening typically used with individuals with dementia, along with other tests selected based on each individual’s cognitive profile and diagnostic needs. The Italian version used in the study [15] was one of the most widely used in Italy at that time and provides normative data on 1,019 elderly individuals aged 65–89; it is an identical translation of the original MMSE [14] and consists of sub-tests assessing: Orientation in time and space, Registration (immediate recall of three words), Attention and Calculation (serial subtraction task or spelling backward), Recall (delayed recall of the previously presented three words), Language (naming objects, repeating a phrase, following verbal and written commands), and Constructional Praxis (copying a complex figure).

The latter part of the evaluation was tailored to each patient, and only the MMSE results were available for all participants. A cohort of 3,055 consecutive individuals arrived for a first assessment (Time 1, T1); from this whole cohort, 531 individuals were further visited a second time (Time 2, T2) and 117 underwent a third follow-up (Time 3, T3). In sum, 117 individuals (70 females) were monitored over time through three consecutive evaluations and this formed the sample of the present study. On average, participants were seen for the second time after one and a half years on average (mean = 1.53 ± 1.64, range = 1–10), while they were seen for the third time after two or three years on average (mean = 2.95 ± 2.11, range = 1–8). See Figures S1 and S2 in the Supplementary Materials for further information about the time intervals between assessments.

The reason for the variability of the time interval between follow-ups depended on many reasons. Although at the initial assessment all individuals were invited for subsequent evaluations, not all of them returned for a re-evaluation. This was due to various intervening factors, for example some opted for different hospitals, others experienced worsening health conditions necessitating hospitalization or residential care. Additionally, some individuals, feeling not so concerned about their condition, did not undergo further assessment. Such circumstances were inevitably out of the experimenters’ control.

At baseline, the mean age of participants was 78.2 ± 7.53 years (range: 45–99), mean education was 5 ± 4.5 years (range: 2–22), and MMSE score was 23.94 ± 3.45 out of 30 (range: 9–30). According to test scores and qualitative evaluation, the sample was characterized by varying degrees of age-related cognitive decline ranging from Subjective Cognitive Decline, or Mild Neurocognitive Disorder, to Major Neurocognitive Disorder [16, 17]. Cognitive efficiency was estimated through the MMSE screening, which was available for all 117 individuals. The MMSE is the standard tool typically used to test residual capacities even in severe patients; it is a global cognitive screening that taps into 11 cognitive areas (temporal orientation, spatial orientation, immediate memory, attention, delayed memory recall, naming, verbal repetition, verbal comprehension, writing, reading, and constructional praxis); the MMSE score was used in our analyses as an index of cognitive efficiency of individuals in the sample. The study was conducted in accordance with the Declaration of Helsinki and under the approval of the Ethical Committee of the School of Psychology at the University of Padua, Italy.

### Statistical analyses

We measured CR by combining education (years of schooling) and occupational activity using the International Standard Classification of Occupations 2008 code (ISCO-08), an internationally supported standard for labour statistics [see also 5]. This system classifies each working activity on a hierarchy structure where lower values indicate more complex jobs (e.g., 9629 corresponds to ‘elementary workers not elsewhere classified’, while 1110 corresponds to ‘legislator’).

**Fig. 1:**
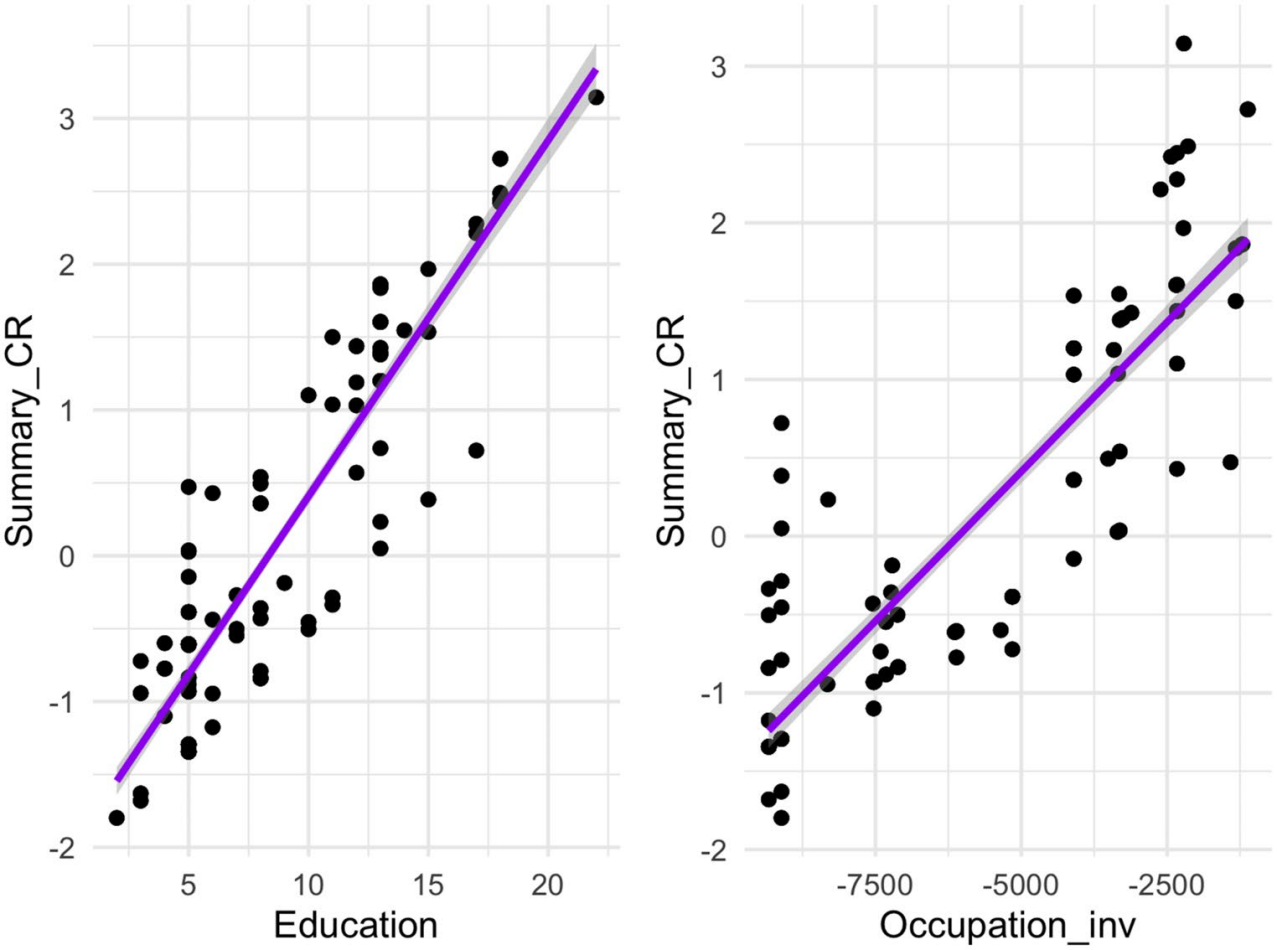
Correlation between CR score and Education, and with the inverse ISCO-08 scores. Both the socio-demographic variables significantly correlated with the CR score

A composite score of CR was obtained by combining years of education with occupation using Principal Component Analysis (PCA) for dimensionality reduction. Education and occupation were both transformed into z-scores before running the PCA. The first component of PCA accounted for 78% of data variance; the CR score resulted from the combination of years at school and occupational activity classified with ISCO-08 (see for a similar approach [16]). After checking whether the CR score was positively correlated with education (Pearson’s *r* = 0.91) and with the inverse transformed values of the ISCO-08 (Pearson’s *r* = 0.86), participants were divided in two groups: “high CR” (*N* = 60 individuals) and “low CR” (*N* = 57), following similar approaches used previously [2, 17]; Fig. 1 below.

Table 1 shows the results of age comparison between the group with high CR and the group with low CR at the three assessment times. No differences were found between the two CR groups as concerns sex [high CR = 26 males and 33 females; low CR = 21 males and 37 females; (Chi-square = 0.46, *p* = 0.49)], time interval across assessments in years [(mean high CR = 1.64 ± 0.69; mean low CR = 1.59 ± 0.67, (*B* = -0.04, *p* = 0.61)], and the number of physical comorbidities (mean high CR = 1.25 ± 1.16; mean low CR = 1.16 ± 1.19 (*B* = -0.09, *p* = 0.47)].

**Table 1:** Age of participants in the high and low CR group at the different assessments. The two groups did not differ significantly by age across assessments

	High CR *N* = 60	Low CR *N* = 57	High CR vs. Low CR	
	Mean (SD)	Mean (SD)	t	*p*-value
Age at T1	74.08 (8.45)	75.72 (6.18)	-1.19	0.23
Age at T2	75.67 (8.46)	77.64 (5.82)	-1.42	0.15
Age at T3	77.25 (8.63)	78.83 (6.11)	-1.14	0.25

In the first regression model, the performance on global cognitive screening was analyzed across the three assessment times (Model 1) to evaluate the general trend of cognitive performance in the whole sample and included the global cognitive score as dependent variable and time of assessment as predictor of interest. The longitudinal trajectory was also observed across the two CR levels (Model 2), which included the interaction between CR and time of assessment (three levels) to see whether the cognitive performance followed different trajectories in relation to the CR level. In addition to the output resulting from the model, the Akaike Information Criterion (AIC) was also computed to test model likelihood and AICs were statistically compared. All statistical analyses were run with the R Software (version 4.2.2).

## Results

**Fig. 2:**
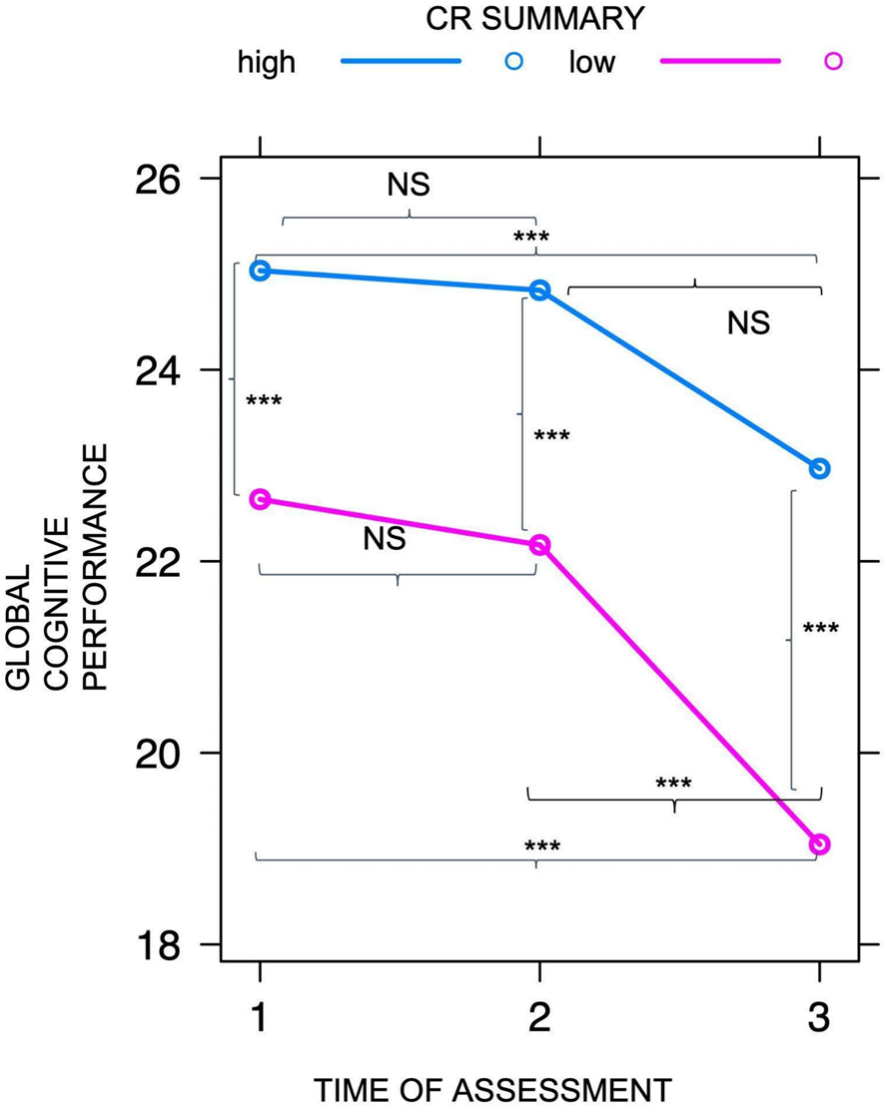
Trajectories of cognitive performance in the two CR groups across time. The three times of assessment are reported on the x-axis. The score of global cognitive performance (MMSE) is reported on the y-axis. The two lines indicate the different levels of CR based on education and occupational complexity. NS = Not Significant difference between conditions

Overall, the cognitive performance of the whole sample (Model 1) worsened from T1 to T3 (B = -3.04, *p* < 0.001), but this change was mainly due to the difference between T2 and T3 (B = -2.27, *p* < 0.001) whereas no significant decrease was found between T1 and T2 (B = -0.76, *p* = 0.17). However, taking CR into account in Model 2, an improvement was found in the model fit (AIC of Model 1 = 2035; AIC of Model 2 = 1999; Sum of Square = 745.55, Chi-square *p* < 0.001). Model 2 showed a significant effect of CR on cognitive performance (B = -2.71, *p* < 0.01) and confirmed a decrease of cognitive performance over time from T2 to T3 (B = -1.91, *p* = 0.01). Post-hoc analyses (adjusted with the Tukey method) compared the performance across times (T1, T2, and T3) considering CR level (high vs. low). At T1 individuals with high CR showed higher cognitive efficiency compared to low CR individuals (B = 2.46, *p* = 0.01). This difference was constantly maintained throughout the assessments (T2: B = 2.71, *p* < 0.01; T3: B = 3.47, *p* < 0.01). From T1 to T2, neither group showed significantly different performance (high CR: B = -0.66, *p* = 0.99; low CR: B = -0.91, *p* = 0.84). However, from T2 to T3, while the performance of individuals with low CR declined significantly (B = 2.66, *p* < 0.01), no difference was found in individuals with high CR (B = 1.91, *p* = 0.12). In the latter group a significant decline emerged only considering the longer time, that is between T1 and T3 (B = 2.56, *p* < 0.01). See Fig. 2.

This pattern of results was confirmed in a sub-group of individuals (*N* = 40) whose clinical diagnosis changed across the three times of assessment, showing a conversion towards dementia: their diagnosis went from Subjective Cognitive Decline or Mild Neurocognitive Disorder at T1 and T2, to Major Neurocognitive Disorder at T3. In this sub-group of “converters”, 21 individuals had high CR and 19 low CR. After controlling for age, the trajectory of decline confirmed a significant advantage in terms of cognitive maintenance in high CR individuals. In other words, while from T1 to T2, both groups showed no differences in cognitive performance (high CR: B = -0.61, *p* = 0.98; low CR: B = -0.35, *p* = 0.78), from T2 to T3 only individuals with low CR showed a significant decline (B = 3.33, *p* = 0.02) while in those with high CR no difference was found (B = 2.68, *p* = 0.6), although they had reached Major Neurocognitive Disorder diagnosis.

Figure S3 in the Supplementary Materials shows how results can be observed in relation with previous models [2, 9].

## Discussion

This study aimed to delineate the trajectory of changes over time in elderly individuals with different levels of Cognitive Reserve. We analyzed the data of a cohort of 117 ageing individuals assessed at three consecutive neuropsychological evaluations. According to previous studies, older adults with high CR seem to be able to maintain their cognitive state over time and contrast their decline.

How the trajectory of cognitive decline in older adults varies depending on their level of CR is less understood and, to date, the literature about the role of CR in the trajectory of cognitive decline has not reported consistent results. Some studies have shown that high CR could “hide” or “mask” the accumulation of pathology, but, despite its protective role, a high CR has been hypothesized to lead to a more rapid decline than low CR once dementia has taken hold as an effect of larger accumulation of physiological pathology in the brain [2, 10]. On the other hand, other studies have shown that a high CR does not necessarily lead to accelerated cognitive deterioration. For example, a previous research [18] has tested whether there must be a certain degree of neuropathological burden before CR can influence cognitive trajectories and showed that CR does not predict cognitive changes over time when the pathology is under a certain clinical threshold, but it does when pathology is overt.

Overall, the results of the present study show that, as expected, older adults worsened from T1 to T3 [19] but the trajectory declined significantly only from T2 to T3 showing a non-linear progression. At T1 Individuals with high CR showed higher cognitive efficiency than those with low CR, as expected, but their decline followed a different trajectory: they did not show any acceleration over time, rather they maintained a more stable functioning than those with low CR, whose decline was indeed steeper. This same pattern was confirmed in a subset of participants who, based on the assessment, converted from Subjective Cognitive Decline/Mild Neurocognitive Disorder at T1/T2 to Major Neurocognitive Disorder at T3. These results are in line with previous findings [19] which demonstrated that compared with individuals with low education, older adults with high education showed an advantage at the MMSE at baseline and a slower decline than their counterpart [19]. This advantage of individuals with high CR is also in line with a recent study showing that individuals with more years of schooling can maintain higher efficiency over time than those with lower education [11].

Building on previous research demonstrating how a composite CR may account for more variance than education alone [20], we derived a CR index by combining education and occupation through the lifespan [4, 21]. This index allowed us to demonstrate that high CR individuals not only bear up longer, but their decline, if any, can occur more gradually than individuals with low CR. Notwithstanding the growing amount of literature on CR [22, 23], our findings allow some further considerations: after distinguishing participants in two sub-groups based on their CR, we discovered that they also differed by distinct rates of trajectory of decline, which we did not observe when considering them all together.

Considering the limitation of having the Mini-Mental State Examination [MMSE, 14, 15] as a single test to evaluate participants’ cognitive abilities, it is important to underscore the comprehensive insight that it provides. In fact, despite its low sensitivity to the nuanced cognitive challenges in the early stages of dementia, the MMSE offers a global perspective on cognitive functioning. Using such screening can be sufficiently informative with our high number of participants, and it allows longitudinal comparisons that can track the progression of dementia. Moreover, the MMSE proves to be valuable especially in the severe stages of the condition by offering crucial information on patients’ cognitive state and aiding in the management of their care [24]. Another potential limitation of this study is the lack of neuroanatomical measures to track brain level trajectories and so whether variations in the levels of pathology and/or presence of biomarkers could influence the effect of CR remains unanswered. In this study, structural neuroimaging measures (CT scan or MRI) were incorporated only in the clinical diagnostic process, helping in the characterization of each participant’s neuropsychological profile.

In conclusion, this study offers robust empirical evidence supporting the influential role of CR in modulating trajectories of decline and highlights the profound clinical implications of CR, indicating that individuals with high CR are likely to experience a more attenuated cognitive decline. This suggests that CR is a buffer against the severity of cognitive deterioration even in more advanced stages of decline, and prompts us to underscore the protective effects of CR throughout the natural ageing process. Our findings pave the way for further research on interventions aimed to enhance CR, encouraging the use of strategies to mitigate age-related cognitive decline.

## Electronic supplementary material

Below is the link to the electronic supplementary material.

Supplementary Material 1

## Data Availability

No datasets were generated or analysed during the current study.
