# Supplementary material for: Cognitive reserve can impact trajectories in ageing: a longitudinal study
Source: Aging Clin Exp Res. 2025 Mar 17;37(1):93. doi: 10.1007/s40520-025-03000-z (PMC11914246; doi:10.1007/s40520-025-03000-z)
Supplement: Supplementary file 1 — Supplementary Material 1 [file 40520_2025_3000_MOESM1_ESM.docx]

**Cognitive Reserve can impact Trajectories in ageing: A longitudinal study**

Sonia Montemurro**^1^**, Raffaella Ida Rumiati**^2^**, Veronica Pucci**^1,3^**, Massimo Nucci**^4^** and Sara Mondini**^1,3^**

**^1^**Department of Philosophy, Sociology, Education and Applied Psychology, University of Padua, Italy

**^2^**Neuroscience, SISSA; Trieste, Italy

**^3^**Human Inspired Technology Centre (HIT), University of Padua, Italy

**^4^**Department of General Psychology, University of Padua, Italy

**
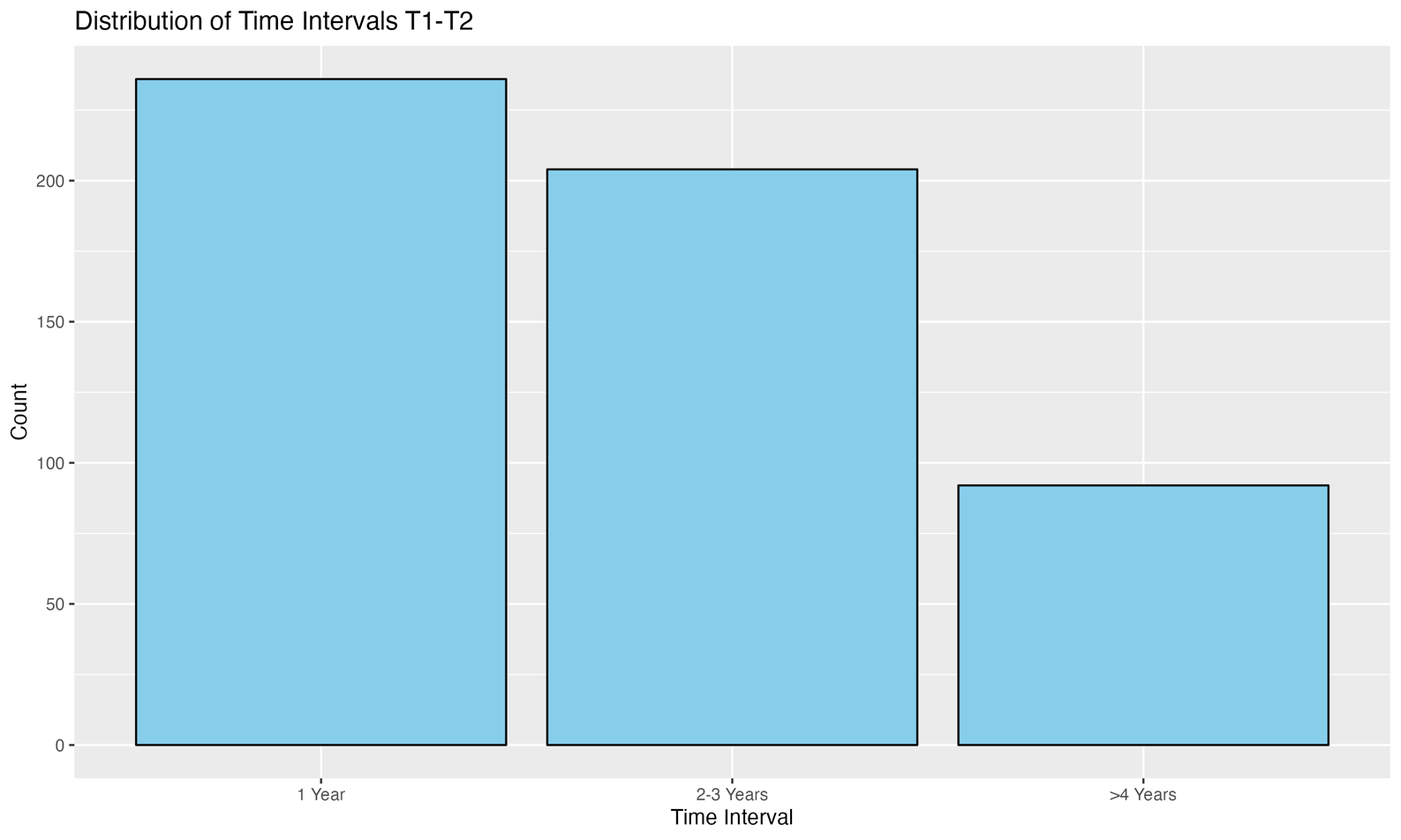
**

**Figure S1. Distribution of older adults between T1 and T2.** On the x-axis the time intervals are reported. On the y-axis the distribution of frequency in the participants between the first assessment (T1) and the second assessment (T2) is reported.


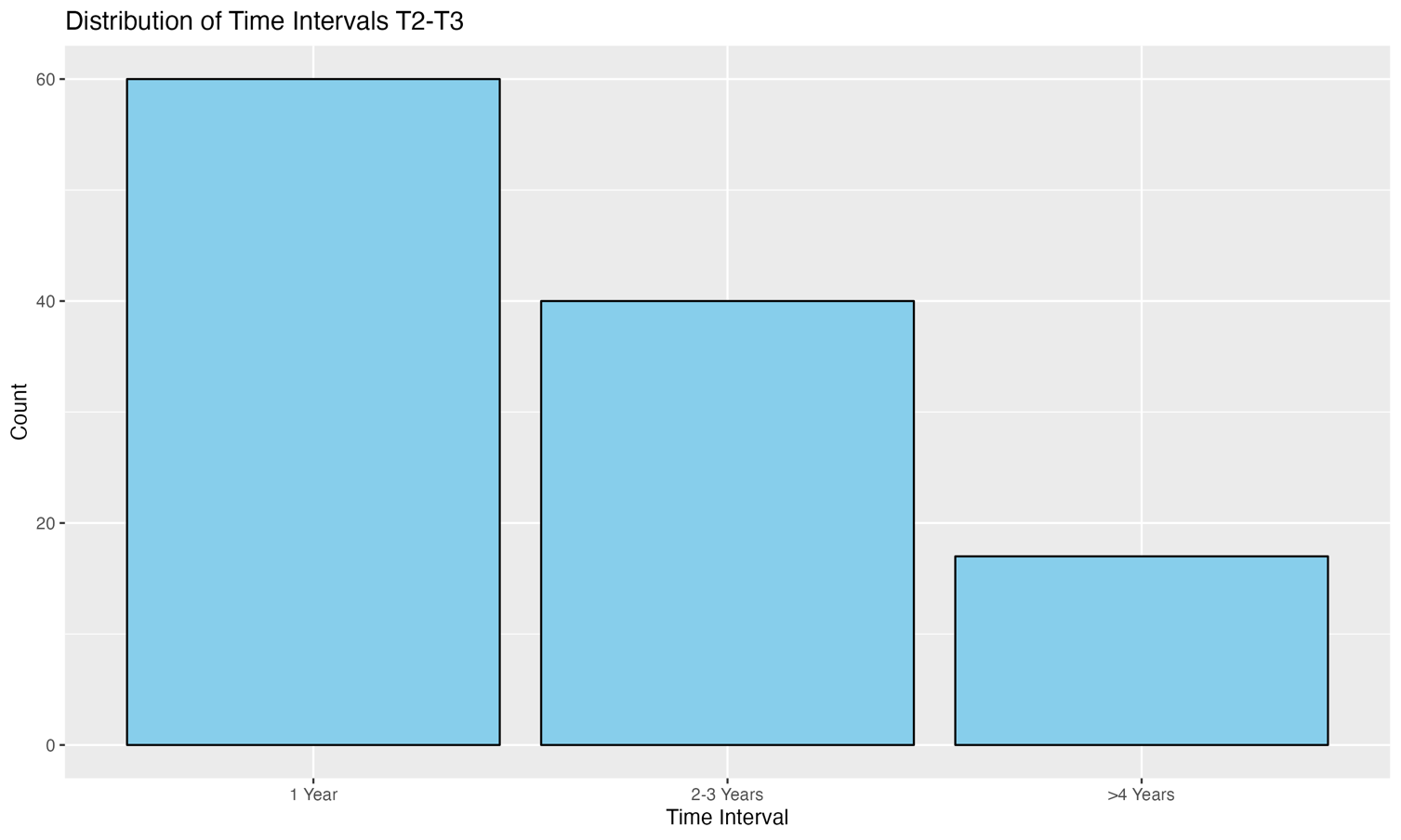


**Figure S2. Distribution of older adults between T2 and T3.** On the x-axis the time intervals are reported. On the y-axis the distribution of frequency in the participants between the first assessment (T2) and the second assessment (T3) is reported.


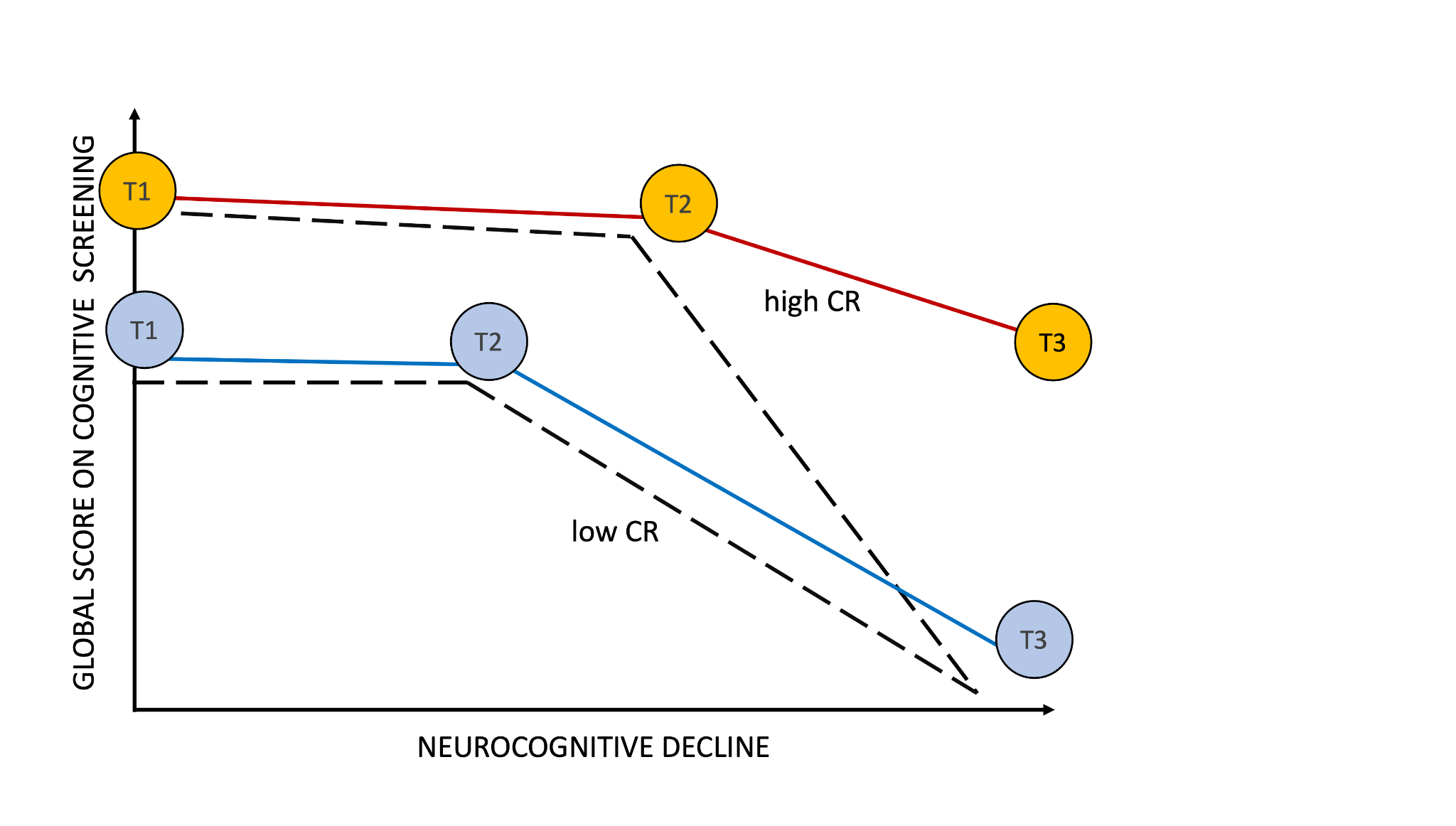


**Figure S4. Representation of results of this study compared with Stern’s model (2009, 2012).** This figure shows the trajectories of cognitive ageing associated with typical dementia symptoms, based on Stern and colleagues’ model (2009, 2012). The circles indicate the three assessment times (T1, T2, T3) of our study. The dotted lines of high and low cognitive reserve are those defined by Stern, while the red and blue coloured lines represent the trajectories of our data, showing a significant decline only in the case of elderly people with low CR. In our study the two groups did not differ by sex, age and time interval between the three assessments.

**Post-hoc analyses**

| **Contrasts** | **Beta** | **Standard Error** | **df** | **t** | **p-value** |
| --- | --- | --- | --- | --- | --- |
| Time_of_Assessment1 high - Time_of_Assessment2 high | 0,67 | 0,76 | 345 | 0,88 | 0,95 |
| Time_of_Assessment1 high - Time_of_Assessment3 high | 2,57 | 0,76 | 345 | 3,39 | 0,01 |
| Time_of_Assessment1 high - Time_of_Assessment1 low | 2,46 | 0,76 | 345 | 3,23 | 0,02 |
| Time_of_Assessment1 high - Time_of_Assessment2 low | 3,38 | 0,77 | 345 | 4,41 | <0.001 |
| Time_of_Assessment1 high - Time_of_Assessment3 low | 6,05 | 0,77 | 345 | 7,88 | <0.001 |
| Time_of_Assessment2 high - Time_of_Assessment3 high | 1,90 | 0,75 | 345 | 2,52 | 0,12 |
| Time_of_Assessment2 high - Time_of_Assessment1 low | 1,79 | 0,76 | 345 | 2,36 | 0,17 |
| Time_of_Assessment2 high - Time_of_Assessment2 low | 2,71 | 0,76 | 345 | 3,55 | 0,01 |
| Time_of_Assessment2 high - Time_of_Assessment3 low | 5,38 | 0,76 | 345 | 7,04 | <0.001 |
| Time_of_Assessment3 high - Time_of_Assessment1 low | -0,11 | 0,76 | 345 | -0,14 | 1,00 |
| Time_of_Assessment3 high - Time_of_Assessment2 low | 0,81 | 0,76 | 345 | 1,06 | 0,90 |
| Time_of_Assessment3 high - Time_of_Assessment3 low | 3,48 | 0,76 | 345 | 4,55 | <0.001 |
| Time_of_Assessment1 low - Time_of_Assessment2 low | 0,92 | 0,77 | 345 | 1,19 | 0,84 |
| Time_of_Assessment1 low - Time_of_Assessment3 low | 3,58 | 0,77 | 345 | 4,65 | <0.001 |
| Time_of_Assessment2 low - Time_of_Assessment3 low | 2,67 | 0,77 | 345 | 3,45 | 0,01 |

**Table S1. Post-hoc analyses.** The table reports the statistics related to post-hoc comparisons related to the regression Model 2. The Tukey method was applied for multiple comparison correction.

|  | **T1** | **T2** | **T3** |
| --- | --- | --- | --- |
| *Major NCD* | *58* | 72 | 90 |
| *Mild NCD* | *41* | 27 | 16 |
| *SCD* | *18* | 18 | 11 |

**Table S1. Distribution of diagnostic groups at the three times of assessment.** Major NCD = Major Neurocognitive Disorder, Mild NCD = Mild Neurocognitive Disorder, SCD = Subjective Cognitive Decline.
